# Coverage for Continuous Glucose Monitoring for Individuals with Type 2 Diabetes Treated with Nonintensive Therapies: An Evidence-Based Approach to Policymaking

**DOI:** 10.1089/dia.2023.0268

**Published:** 2023-10-12

**Authors:** Grazia Aleppo, Irl B. Hirsch, Christopher G. Parkin, Janet McGill, Rodolfo Galindo, Davida F. Kruger, Carol J. Levy, Gregory P. Forlenza, Guillermo E. Umpierrez, George Grunberger, Richard M. Bergenstal

**Affiliations:** ^1^ Division of Endocrinology, Metabolism and Molecular Medicine, Feinberg School of Medicine Northwestern University, Chicago, Illinois, USA.; ^2^ University of Washington, Seattle, Washington, USA.; ^3^ CGParkin Communications, Inc., Henderson, Nevada, USA.; ^4^ Division of Endocrinology, Metabolism and Lipid Research, Washington University in St. Louis, School of Medicine, St. Louis, Missouri, USA.; ^5^ Lennar Medical Center, UMiami Health System, Jackson Memorial Health System, University of Miami Miller School of Medicine, Miami, Florida, USA.; ^6^ Division of Endocrinology, Diabetes, Bone & Mineral, Henry Ford Health System, Detroit, Michigan, USA.; ^7^ Division of Endocrinology, Diabetes, and Metabolism, Icahn School of Medicine at Mount Sinai, New York, New York, USA.; ^8^ Division of Pediatric Endocrinology, Department of Pediatrics, Barbara Davis Center, University of Colorado Denver, Aurora, Colorado, USA.; ^9^ Division of Endocrinology, Metabolism Emory University School of Medicine, Grady Memorial Hospital, Atlanta, Georgia, USA.; ^10^ Grunberger Diabetes Institute, Bloomfield Hills, Michigan, USA.; ^11^ International Diabetes Center at Park Nicollet, HealthPartners Institute, Minneapolis, Minnesota, USA.

**Keywords:** Type 1 diabetes, Type 2 diabetes, Continuous glucose monitoring, Basal insulin, Noninsulin medications, HbA1c

## Abstract

Numerous studies have demonstrated the clinical benefits of continuous glucose monitoring (CGM) in individuals with type 1 diabetes (T1D) and type 2 diabetes (T2D) who are treated with intensive insulin regimens. Based on this evidence, CGM is now a standard of care for individuals within these diabetes populations and widely covered by commercial and public insurers. Moreover, recent clinical guidelines from the American Diabetes Association and American Association of Clinical Endocrinology now endorse CGM use in individuals treated with nonintensive insulin regimens. However, despite increasing evidence supporting CGM use for individuals treated with less-intensive insulin therapy or noninsulin medications, insurance coverage is limited or nonexistent. This narrative review reports key findings from recent randomized, observational, and retrospective studies investigating use of CGM in T2D individuals treated with basal insulin only and/or noninsulin therapies and presents an evidence-based rationale for expanding access to CGM within this population.

## Introduction

An estimated 34.5 million people in the United States have type 2 diabetes (T2D),^1^ the majority of whom are treated with noninsulin medications.^2^ An estimated 95% of individuals with T2D are treated with basal insulin only, noninsulin medications, and/or lifestyle interventions.^3–5^

Despite the introduction of new diabetes medications and innovative glucose monitoring technologies, almost half of all diabetes patients have poor glycemic control.^2,6–10^ As reported in the NHANES data, the percentage of adult NHANES participants with diabetes who achieved HbA1c levels of <7.0% declined from 57.4% in 2015 to 50.5% in 2018.^2^

Early landmark studies have demonstrated that persistent hyperglycemia results in long-term microvascular and macrovascular complications of diabetes.^11–14^ Although often not considered, severe hypoglycemia is a significant burden for T2D individuals treated with insulin^15^ and oral antihyperglycemic medications,^16^ particularly for older individuals, many of whom have underlying heart disease.^17^

Frequent monitoring of glucose levels is a critical component of safe and effective diabetes self-management. Although blood glucose monitoring (BGM) with meter and test strips remains the most common method for glucose measurement, studies have shown that adherence to BGM is low.^18–22^ As observed in a large cross-sectional study of 5104 individuals, adherence rates for prescribed BGM were as low as 44% for type 1 diabetes (T1D) adults and 24% for T2D adults.^22^ In a large, 12-month retrospective analysis of 1,329,061 Medicare beneficiaries with T1D and T2D treated with intensive insulin therapy (IIT), investigators reported that 38.14% of beneficiaries did not follow their prescribed glucose monitoring and an additional 35.42% had no record of ever obtaining glucose monitoring supplies.^20^ In a Swedish cross-sectional survey, the most common reasons for nonadherence reported were lack of time, not remembering to test, self-consciousness when testing in public, and the pain/discomfort of fingersticks.^19^

An increasing number of individuals with T1D and intensively managed T2D have transitioned from BGM to continuous glucose monitoring (CGM). Unlike BGM, which provides only point-in-time glucose values, current CGM devices automatically transmit real-time glucose data to users' smartphone or handheld receiver. Users have immediate access to viewing their current glucose values, recent trends, and rate-of-change trend arrows that indicate the direction and velocity of changing glucose, which can be easily and inconspicuously done in public. For added safety, these devices feature alarms and alerts that warn users about current and/or impending severe hypoglycemic (SH) and hyperglycemic events. This is particularly important to individuals with recurrent nocturnal hypoglycemia.

Retrospective analysis of overnight data enables users and their clinicians to adjust therapy to avoid SH events. CGM sensors last 7–14 days (and up to 180 days for implantable sensors), greatly reducing the patient burden associated with capturing these data relative to BGM.

Numerous clinical trials and real-world observational and prospective studies have proved the safety and effectiveness of CGM in individuals with T1D and T2D who are treated with IIT.^23–36^ Based on this evidence, CGM is now a standard of care for individuals within these diabetes populations.^37–39^

Despite a growing body of evidence supporting CGM use among people with T1D and T2D treated with IIT, commercial and public insurance coverage for CGM use in individuals using basal insulin only, or no insulin at all is limited, particularly within populations with lower socioeconomic status and racial/ethnic minority communities, which are disproportionately affected by diabetes.^40,41^ For example, among the 40 state in which Medicaid programs provide coverage, a 2022 report found that 27 currently cover CGMs only for individuals with T1D and T2D treated with IIT.^42^ In this narrative review, we report findings from current randomized and observational/retrospective studies investigating use of CGM in T2D individuals treated with basal insulin only and/or noninsulin therapies and present a rationale for expanding access to CGM within this population.

## Persistent CGM Use in Nonintensively Treated T2D

Both randomized and prospective/retrospective studies have demonstrated significant glycemic improvement, reductions in diabetes-related events and hospitalization rates, and cost benefits of persistent CGM use by individuals with T2D who are treated with basal insulin only, basal plus noninsulin medications, and noninsulin medications without insulin (Table 1). The following is a summary of the major findings from these studies.

**Table 1.** Improvements in Key Outcomes from Randomized and Prospective/Retrospective Studies of Persistent Continuous Glucose Monitoring Use

Study	Study design	Therapies	Outcomes			
			HbA1c	CGM metrics	Events or cost^a^	Psych, QoL or behavior
Persistent use						
Aronson et al.^43^	Randomized	Noninsulin	X	X	—	X
Davis et al.^44^	Randomized	Basal only, basal+noninsulin, post hoc analysis of Mobile study (Martens et al.)	—	X	—	—
Bao et al.^45^	Randomized	Basal only, basal+noninsulin	X	X	—	—
Martens et al.^46^	Randomized	Basal only, basal+noninsulin	X	X	—	—
Aleppo et al.^47^	Randomized	Basal only, basal+noninsulin	X	X^b^	—	—
Wada et al.^48^	Randomized	Noninsulin	X	X	—	X
Welsh et al.^49^	Prospective	Insulin treated, noninsulin	X	—	—	—
Dowd et al.^50^	Retrospective	Intensive insulin, noninsulin	—	X	—	—
Chesser et al.^51^	Pilot Interventional	Basal only, basal+noninsulin, noninsulin	—	—	—	X
Crawford et al.^53^	Retrospective	Basal only	—	X	—	—
Norman et al.^54^	Retrospective	Intensive insulin, basal only, basal+noninsulin	X	—	X	—
Guerci et al.^55^	Retrospective	Basal only, basal+noninsulin	—	—	X	—
Shields et al.^56^	Retrospective	Intensive insulin, basal only, basal+noninsulin	X	—	—	—
Carlson et al.^57^	Retrospective	Basal+noninsulin	X	—	—	—
Grace et al.^58^	Prospective	Basal only, basal+noninsulin, noninsulin	X	X	—	—
Elliot et al.^59^	Retrospective	Basal+noninsulin	X	—	—	—
Wright et al.^60^	Retrospective	Basal+noninsulin, noninsulin	X	—	—	—
Miller et al.^61^	Retrospective	Basal only	—	—	X	—
Norman et al.^62^	Retrospective	Intensive insulin, basal only, basal+noninsulin	X	—	—	—

The table outlines studies where rtCGM or isCGM was used continuously, without interruption during the study period.

^a^ Hypoglycemia, other acute events, hospitalizations/ER visits.

^b^ Glycemic control deteriorated when CGM was discontinued.

—, Metric was not measured, or change was not significant.

X indicates improvement.

CGM, continuous glucose monitoring; ER, emergency room; is-CGM, intermittently scanned-CGM; rtCGM, real-time CGM; QoL, quality of life.

### Randomized trials

Aronson et al.^43^ investigated the impact of intermittently scanned CGM (isCGM) on glycemic control and patient-reported outcomes among 116 adults with T2D (HbA1c 8.6% ± 1.1%) who were treated with noninsulin therapies.^43^ Participants were randomized 1:1 to isCGM use with diabetes self-management education (CGM+DSME) or DSME without isCGM. Among the 99 participants who completed the study at 16 weeks, time in range (TIR) was significantly greater in the CGM+DSME arm (76.3% ± 17.4%) compared to DSME arm (65.6% ± 22.6%) (*P* < 0.01), with lower time above range (TAR) (21.2% ± 18.1% vs. 30.7% ± 24.5%, *P* = 0.037, respectively). The CGM+DSME group also experienced significantly greater HbA1c reductions than the DSME group (−0.9% ± 0.9% vs. −0.5% ± 0.9%, *P* = 0.03, respectively). Glucose monitoring satisfaction scores (Glucose Measurement Satisfaction Survey) were also significantly improved in the CGM+DSME arm (0.6 ± 0.5 vs. 0.0 ± 0.5, *P* < 0.01, respectively).

Davis et al.^44^ conducted a post hoc analysis of the MOBILE cohort to determine if patients with the poorest glycemic control would benefit from real-time CGM (rtCGM) use compared with BGM.^44^ Investigators divided patients into four subgroups based on their baseline HbA1c: ≥8.5%–9.0%, ≥9.0%–9.5%, ≥9.5%–10%, and ≥10.0%. Within the full cohort, rtCGM users experienced a larger decrease from baseline HbA1c (1.08%) compared with BGM use (0.64%), with the greatest reductions seen in patients with ≥10.0% at baseline (2.07% vs. 0.4%).

Bao et al.,^45^ in a sub-analysis of the MOBILE study, assessed the impact of rtCGM use in older adults age ≥65 years (*n* = 42) compared with younger patients (*n* = 133).^45^ The mean change in HbA1c from baseline was −1.08% in the older CGM group compared with −0.38% among older BGM users, with an adjusted mean difference of 0.65%. Significant increases in TIR (70–180 mg/dL) were observed in the older rtCGM and BGM groups (19%, *P* = 0.01 and 12%, *P* = 0.003, respectively). The mean difference in HbA1c between treatment groups was −0.35% in the younger study groups.

Martens et al.^46^ in the recent randomized MOBILE study, assessed the effects of CGM use in 175 T2D adults who were treated with basal insulin with or without noninsulin medications.^46^ Patients were randomized 2:1 rtCGM (*n* = 116) or traditional BGM (*n* = 59) and followed for 8 months. Baseline HbA1c values in the rtCGM and BGM groups were 9.1% and 9.0%, respectively.

At study end, mean change in HbA1c among rtCGM users was −1.1% compared with −0.5% in the BGM group (*P* = 0.02), with significant increases in percentage of TIR (%TIR) in the rtCGM group (from 40% to 56%) compared with decreases in the BGM group (from 59% to 43%, *P* < 0.001), and significant decreases in percentage of TAR (%TAR >250 mg/dL) compared with the increases in the BGM group (−15 vs. 2, *P* < 0.001). Importantly, exploratory subgroup analyses suggested that the HbA1c improvements were present across the age range of 33 to 79 years and the baseline HbA1c range of 7.1% to 11.6%.

Aleppo et al.^47^ in this multicenter study, evaluated the effect of discontinuing rtCGM after 8 months of use.^47^ In Phase 1, patients had initially been randomized to either rtCGM or BGM for 8 months. In Phase 2, the BGM group continued to use BGM (*n* = 57) and rtCGM users were randomized to continue (*n* = 53) or discontinue use (*n* = 53).

All study groups were then followed for another 6 months. In the group that discontinued CGM, mean %TIR had increased from 38% to 62% during the 8 months using rtCGM. Six months after discontinuing rtCGM, %TIR decreased to 50% at 14 months (*P* = 0.01). Mean baseline HbA1c values starting Phase 2 were 7.9% in the discontinued rtCGM group, 8.2% in the continued rtCGM group, and 8.4% in the BGM group. Mean HbA1c increased to 8.2% after discontinuing CGM (*P* = 0.06), whereas HbA1c in patients who continued rtCGM decreased to 8.1% HbA1c, and in the BGM group increased to 8.5% at 14 months.

Wada et al.^48^ in this 24-week, multicenter, open-label, randomized parallel-group study, randomly assigned 93 adult patients with noninsulin-treated T2D (1:1) to isCGM (*n* = 49) or BGM (*n* = 51) use.^48^ Change in HbA1c in each group was assessed. Baseline HbA1c levels were similar between the isCGM and BGM groups (7.83% and 7.84%, respectively). HbA1c was significantly decreased from baseline in the isCGM group at 24 weeks (−0.46%, *P* < 0.001), but not the BGM group (−0.17%, *P* = 0.124); a significant between-group difference was also observed (−0.29%, *P* = 0.022). Treatment satisfaction scores obtained using the Diabetes Treatment Satisfaction Questionnaire were also significantly improved, along with the mean glucose levels, glycemic variability, and time in hyperglycemia compared with the BGM group.

### Prospective/retrospective studies

Welsh et al.^49^ examined the glycemic effects of providing no-cost rtCGM to underinsured patients with T2D in a community setting.^49^ In this interim analysis, 32 individuals who were insulin-treated (*n* = 18) and noninsulin-treated (*n* = 14) with 9.9% baseline HbA1c were followed for 6 months. Among all patients, investigators observed a significant decrease in HbA1c from baseline at 3 months (−2.7%), which sustained at 6 months (−2.8%). Differences between HbA1c levels between insulin-treated and noninsulin-treated patients were insignificant (7.1% vs.7.2%).

Dowd et al.^50^ conducted a retrospective analysis of uploaded data from 33,685 U.S.-based Dexcom G6 rtCGM users who self-identified as either T1D (*n* = 26,706) or T2D treated with noninsulin therapies (*n* = 6979) to assess participants' glycemic metrics and determine how they used their alerts and other rtCGM features.^50^ T2D versus T1D patients spent more time at glucose levels 70–180 mg/dL (70.8% vs. 52.1%, respectively) with less time <70 mg/dL (0.8% vs. 2.4%, respectively) and >180 mg/dL (28.5% vs. 45.5%, respectively). A larger proportion of T1D than T2D patients continued to upload their rtCGM data at 1 month (73.7% vs. 53.6%, respectively) and 2 months (69.9% vs. 48.0%, respectively).

Large proportions of both T2D and T1D patients enabled and customized their glucose alerts. However, a higher proportion of T1D patients utilized the data SHARE feature compared with T2D patients (38.0% vs. 10.0%, respectively) and had more followers compared with T2D patients. Similar proportions of T2D (53.8%) and T1D (59.1%) patients used the CLARITY software throughout the observation period.

Chesser et al.^51^ in this pilot, single-arm, interventional study assessed the usability and feasibility of 12 weeks' use of CGM in nine adolescents and young adults (13–21 years old) with T2D for ≥6 months, HbA1c >7.0%, and treated with basal insulin and/or noninsulin therapy (NIT); seven participants completed.^51^ At 12 weeks, participants reported statistically significant improvement in diabetes-related quality of life (QoL), with the mean Pediatric Quality of Life inventory (PedsQL)^52^ diabetes score increasing from 70 to 75 after using CGM (*P* = 0.026).

Crawford et al.,^53^ in a 12-week observational study of 150 T2D adults treated with nonintensive insulin therapy (NIIT), evaluated the effects of rtCGM use on key glycemic metrics.^53^ Patients received no additional education beyond standard of care. At 12 weeks, rtCGM data revealed significant increases in %TIR in 53 (35%) of patients at all time periods: 12 a.m.–5 a.m., from 96.7% to 98.2%; 5 a.m.–12 p.m., from 66% to 94.0%; 12 p.m.–5 p.m., from 67.6% to 88.1%; and 5 p.m.–12 a.m., from 63.3% to 83.9% (all *P* < 0.05). Significant (*P* < 0.05) reductions in peak glucose at all time periods were also observed.

Norman et al.,^54^ in the retrospective analysis of administrative claims data in the Optum Research Database (ORD), investigated the impact of rtCGM on diabetes-related medical costs within the T2D population following ≥6 months of rtCGM use.^54^ Changes in diabetes-related health care resource utilization costs were expressed as per-patient-per-month (PPPM) costs. Within the cohort, 454 (80%) patients were treated with IIT, 58 (10%) were treated with NIIT, and 59 (10%) were treated with NIT. Results showed that the average PPPM for diabetes-related medical costs decreased by $424 (*P* = 0.035) after initiating rtCGM. Reductions in inpatient hospital admissions (−0.006 PPPM, *P* = 0.057) and in total hospital admissions (−0.042 PPPM, *P* = 0.139) were also observed.

Guerci et al.^55^ conducted a retrospective analysis of the French national Système National des Données de Santé reimbursement claims database and identified 5933 individuals with T2D who had initiated isCGM between August 1, 2017, and December 31, 2018; 78.9% of subjects were treated with basal insulin and other antihyperglycemic medications.^55^ Claims data for the 12 months before, and up to 24 months after isCGM initiation, were analyzed to identify hospitalizations for adverse diabetes events (ADEs), including SH events, diabetic ketoacidosis (DKA), coma, and hyperglycemia-related admissions. Investigators reported that 2.01% of subjects had experienced at least one hospitalization for any ADE in the year before isCGM initiation, compared to 0.75% at 1 year and 0.60% at 2 years of isCGM use. During the first year of isCGM, there were 75% fewer DKA admissions and a 44% reduction in SH admissions. These improvements persisted after 2 years, with a further 43% reduction in DKA rates.

Shields et al.^56^ utilized data from 13 American Medical Group Association member health systems and multispecialty medical groups to assess the effects of rtCGM on changes in HbA1c in a mixed cohort of 458 patients treated with IIT (*n* = 343 [IIT]), NIIT (*n* = 51 [NIIT]), or NIT (*n* = 64 [NIT]).^56^ Investigators observed that individuals with a baseline HbA1c >7.5 (*n* = 306) showed an average decrease of 0.9% (*P* < 0.001), which varied by treatment regimen: IIT −0.76% (*P* < 0.001); NIIT −1.59% (*P* < 0.001; and NIT −1.13% (*P* < 0.01). HbA1c changes in individuals with ≤7.5% baseline HbA1c were not statistically significant.

Similar findings were reported by Carlson et al.^57^ in a U.S. chart review of 100 T2D adults managed with NIIT.^57^ After 3 to 6 months of isCGM use, the greatest reductions in HbA1c were observed in patients with >9.0% HbA1c at baseline (−1.7%, *P* < 0.0001).

Grace and Salyer^58^ conducted a 6-month, prospective, interventional, single-arm study that investigated use of rtCGM in 38 T2D adults; 58% were treated with noninsulin medications.^58^ At 6 months, rtCGM use was associated with a 3.0% decrease in the mean baseline HbA1c (from 10.1% to 7.3%, *P* < 0.001) and significant increases in %TIR (from 57.0% to 72.2%, *P* < 0.001). All participants maintained targets for hypoglycemia (<4% at 70 mg/dL, <1% at 54 mg/dL). Individuals treated with ≤1 medication showed the greatest increase in %TIR (17.5, *P* = 0.02) compared with those treated with ≥2 medications (13.9, *P* = 0.017) and greatest decrease in %TAR >180 mg/dL (−17.0, *P* = 0.005 vs. −13.9, *P* = 0.024, respectively).

Elliot et al.,^59^ in a recent chart review of 91 poorly controlled T2D adults (9.4% baseline HbA1c) treated with basal insulin therapy, reported significant reductions in HbA1c (−0.8%, *P* < 0.0001) after 3 to 6 months of isCGM use.^59^ Results from a subgroup analysis that compared patients with baseline HbA1c of <9.0% and >9.0% showed clinically significant reductions in the higher HbA1c group (−1.6%, *P* < 0.0001).

Wright et al.,^60^ in this retrospective, observational study, conducted an analysis of the Explorys commercial databases to assess the impact of CGM in 1034 poorly controlled T2D adults (baseline HbA1c 10.1%) treated with basal insulin (*n* = 306) or NIT (*n* = 728).^60^ At 6 months following acquisition of the isCGM sensor, investigators observed significant reductions in HbA1c in the basal-insulin group (−1.1%, *P* < 0.001) and noninsulin treatment group (−1.6%, *P* < 0.001). Individuals with the highest baseline HbA1c (≥12.0%, *n* = 181) showed the greatest reduction (−3.7%, *P* < 0.001).

Miller et al.,^61^ using a similar study design, analyzed data from the MarketScan™ administrative claims database to assess the effects of isCGM on rates of all-cause hospitalizations (ACHs) and ADEs in a larger cohort of T2D adults (*n* = 10,282) treated with NIIT or noninsulin medications.^61^ During the 6-month observation period, investigators reported significant reductions in ACHs, from 0.177 to 0.151 events/patient/year (*P* = 0.002). The rate of ADE decreased from 0.076 to 0.052 events/patient/year (*P* < 0.001). These decreases were significant in both the insulin-treated and noninsulin-treated patients (−0.040, *P* < 0.001 and −0.014, *P* = 0.015 events/patient/year, respectively).

Norman et al.^62^ in an earlier analysis of the ORD, assessed the glycemic effects of CGM use (rtCGM or isCGM) on glycemic control compared with BGM within a cohort of 82,983 T2D patients treated with IIT, NIIT, or NIT.^62^ Following ≥6 months of CGM use, significant reductions in HbA1c were observed with any CGM and rtCGM versus BGM (−0.46 and −0.72 vs. −0.09, respectively, *P* < 0.001). HbA1c reductions were significant in CGM versus BGM users in the IIT group (any CGM, −0.38%; rtCGM, −0.68%, *P* < 0.001) and NIT group (any CGM, −0.67%, *P* < 0.001; and rtCGM, −0.87, *P* = 0.008). Investigators also reported significantly higher percentages of CGM versus BGM users achieved ≥1.0 HbA1c reductions in the IIT and NIT groups.

## Intermittent CGM Use in Nonintensively Treated T2D

In addition to the glycemic benefits observed with persistent CGM use, studies have also demonstrated the utility of intermittent use in modifying user behaviors and improving their understanding of their diabetes and importance of adherence to prescribed nonintensive treatment regimens (Table 2). The following is a summary of the major findings from these studies of intermittent CGM use. Many of these studies involved older CGM technologies.

**Table 2.** Improvements in Key Outcomes from Randomized and Prospective/Retrospective of Intermittent Continuous Glucose Monitoring Use

Study	Study design	Therapies	Outcomes				
			HbA1c	CGM metrics	Events*^a^* or cost	Weight or BMI	Psych, QoL or behavior
Intermittent use							
Moon et al.^63^	Randomized	Noninsulin	X	—	—	—	—
Cox et al.^64^	Randomized	Noninsulin	X	—	—	—	X
Fonda et al.^66^	Randomized	Basal only, basal+noninsulin, noninsulin		—	X	—	—
Vigersky et al.^65^	Randomized	Basal only, basal+noninsulin, noninsulin	X	—	—	—	—
Yoo et al.^67^	Randomized	Basal only, basal+noninsulin, noninsulin	X	—	—	X	
Porter et al.^68^	Prospective	Basal only, basal+noninsulin	—	—	—	X	X
Bergenstal et al.^69^	Retrospective	Basal only, basal+noninsulin, noninsulin	X	—	—	—	X
Majithia et al.^71^	Prospective	Basal only, basal+noninsulin, noninsulin	X	X	—	X	
Polonsky et al.^72^	Retrospective	Basal only, basal+noninsulin, noninsulin	—	—	—	—	X
Dixon et al.^74^	Retrospective	Basal only, basal+noninsulin, noninsulin	X	—	—	—	—

The table illustrates studies where CGM was only worn on an intermittent basis, not continuously.

^a^ Hypoglycemia, other acute events, hospitalizations/ER visits.

—, Metric was not measured, or change was insignificant.

X indicates improvement.

BMI, body mass index.

### Randomized controlled trials

Moon et al.^63^ conduced a multicenter, randomized prospective study to investigate the efficacy of intermittent, short-term rtCGM use in 61 T2D adults who were poorly controlled with noninsulin medications.^63^ Patients were randomly assigned to one of three treatment groups: one session of rtCGM (group 1) or two sessions of rtCGM with a 3-month interval between sessions (group 2) and a control group. All participants used blinded rtCGM for up to 6 days before randomization. Among the 48 patients who completed the study (baseline HbA1c 8.2%), investigators observed a significant HbA1c reduction in treatment group 1 (−0.60%, *P* = 0.044) and treatment group 2 (−0.64%, *P* = 0.014) compared with the control group at 3 months. At 6 months, only group 2 achieved a significant HbA1c reduction (−0.68%, *P* = 0.018).

Cox et al.^64^ in this randomized clinical trial, compared conventional medication management to medication management in conjunction with a lifestyle intervention using CGM in 30 T2D adults treated with noninsulin therapies and mean 8.8% HbA1c at baseline.^64^ Participants were randomly assigned (1:2) to routine care (RC) or use of rtCGM with four discussion sessions about how to minimize glycemic excursions. At the 5-month follow-up, the rtCGM group showed significant improvements in HbA1c compared with the RC group (from 8.9% to 7.6% vs. 8.8% to 8.7%, respectively, *P* = 0.03). The rtCGM group also showed a reduced need for diabetes medication (*P* = 0.01), reduced carbohydrate consumption (*P* = 0.009), and improved diabetes knowledge (*P* = 0.001), QoL (*P* = 0.01), and diabetes distress (*P* = 0.02) and trended to more empowerment (*P* = 0.05) with no increase in hypoglycemia.

Vigersky et al.^65^ and Fonda et al.^66^ in this randomized controlled trial, assessed the glycemic short- and long-term effects of intermittent rtCGM use in a cohort of 100 T2D adults treated with nonintensive therapies.^65^ The majority of patients (*n* = 60) were treated with oral or noninsulin injectable medications only. Investigators compared the effects of 12 weeks of intermittent rtCGM use with BGM on glycemic control over a 40-week follow-up period. At 12 weeks, there was a significant difference in HbA1c that was sustained during the follow-up period. Investigators observed decreases in HbA1c of 1.0%, 1.2%, 0.8%, and 0.8% in the rtCGM group at weeks, 12, 24, 38, and 52, respectively, compared with reductions of 0.5%, 0.5%, 0.5%, and 0.2% in the BGM group (*P* = 0.04).

The improvements observed in the rtCGM group occurred without a greater intensification of medication compared with those in the BGM group. Within this cohort, Fonda et al. reported that intermittent rtCGM use was a cost-effective diabetes management option, and that frequent use may result in additional cost-effectiveness.^66^

Yoo et al.,^67^ in this early prospective, randomized trial, investigated the potential effects of intermittent rtCGM use compared with BGM on glycemic control, weight, and self-management behaviors in a cohort of 65 T2D adults who were poorly controlled with basal insulin and/or oral hypoglycemic agent therapy (8.0% to 10% HbA1c range at baseline).^67^ Patients were randomly assigned to rtCGM or BGM use and followed for 3 months. rtCGM patients used their device once a month for 3 days (due to the wear time of the sensor). The BGM group continued to test glucose levels ≥4 times/week for 3 months. Medication dosages were not to be changed over the study period. Investigators reported significant HbA1c reductions with rtCGM use compared BGM ((−1.1% vs. −0.4%, respectively, *P* < 0.01).

Significant reductions in weight (*P* = 0.014) and BMI (*P* = 0.008) were observed in the rtCGM group but not the BGM group. There was a significant reduction in total calorie intake in the rtCGM group (from 1858.7 to 1690.0 cal/day, *P* = 0.002). A significant increase in exercise time in rtCGM users compared with BGM users (*P* = 0.02) was also observed.

### Prospective/retrospective studies

Porter et al.,^68^ in this prospective pilot study, examined how use of rtCGM impacts glycemic metrics, weight loss, lifestyle, and patient perspectives on using the sensor among 37 T2D adults not treated with prandial insulin.^68^ Participants were randomized to either lifestyle counseling with two 10-day sessions of rtCGM use in blinded mode (*n* = 22) or four 10-day sessions of rtCGM use in unblinded mode but with no counseling (*n* = 15); 13 rtCGM participants completed the study. At 24 weeks, 6 (46.0%) reported weight loss of ≥10 lbs, 11 (84.6%) reported they were motivated to increase physical activity and excluded or eliminated certain foods as a result of rtCGM use, 12 (92.3%) reported they would wear rtCGM on a regular basis, and 13 (100%) reported that use of rtCGM contributed to self-care.

Bergenstal et al.^69^ in this retrospective analysis, evaluated patient satisfaction with CGM use in 594 Onduo/Virtual Diabetes Clinic (VDC) participants.^69^ Satisfaction was assessed using the CGM satisfaction questionnaire.^70^ The reported CGM satisfaction score was 4.5 out of 5. Most respondents (94.7%) agreed/strongly agreed that CGM use improved their understanding of the impact of CGM on eating (97.0%), and that it increased their knowledge about diabetes (95.7%) and helped improve diabetes control when not wearing the sensor (79.4%). HbA1c decreased from 7.7% ± 1.6% to 7.1% ± 1.2% (*P* < 0.001; 10.2 months). These data suggest that it is feasible to provide CGM directly to individuals with T2D through a VDC without in-office training. Importantly, a subgroup analysis revealed significant reductions in HbA1c among both the insulin and noninsulin user groups with a baseline HbA1c ≥8.0%, −1.5% ± 2.1%, and −2.0% ± 1.7%, respectively (both *P* < 0.001).

Majithia et al.^71^ conducted the Onduo VDC program, which is a telehealth program for people with T2D, and the eligibility criteria for participating in the VDC were age ≥18 years and ≥8% HbA1c at program entry. The program combines use of a mobile app, remote lifestyle coaching, connected devices, and live video consultations with board-certified endocrinologists. In this prospective single-arm study, investigators evaluated glycemic outcomes associated with rtCGM use by program participants for 4 months in 55 program members.^71^ Participants were asked to use their rtCGM sensor intermittently over the course of 4 months, wearing a total of six 10-day sensors. At the end of the observation period, HbA1c levels decreased significantly (−1.6%, *P* < 0.001).

When stratified by baseline HbA1c (8.0%–9.0% [*n* = 36] and >9.0% [*n* = 19]), HbA1c decreased by 1.2% (*P* < 0.001) and 2.4% (*P* < 0.001), respectively. %TIR increased by 10.2%, from 65.4% to 75.5% (*P* = 0.02). %TAR (>180 mg/dL) and %TAR (>250 mg/dL) decreased by 7.2% (*P* = 0.005) and 3.0% (*P* = 0.01), respectively. There was no change in %TBR (<70 mg/dL). Investigators reported significant decreases in weight (−9.0 lbs, *P* < 0.001) and improvements in systolic blood pressure (*P* = 0.04), total cholesterol (*P* < 0.001), low-density lipoprotein cholesterol (*P* < 001), and triglycerides (*P* = 0.008).

Polonsky et al.^72^ used the 17-item Diabetes Distress Scale (DDS17)^73^ to evaluate change in diabetes distress among 228 Onduo/VDC participants who reported moderate distress (score 2.0–2.9) or high distress (score ≥3.0) on the time of enrollment. Significant reductions in overall distress from 3.0 ± 0.8 at baseline to 2.5 ± 0.9 (*P* < 0.001) were reported by participants at an average of 6 months of follow-up. Participants who used intermittent rtCGM (*n* = 77) versus nonusers (*n* = 151) reported significantly greater reductions in overall distress (*P* = 0.012) and regimen-related distress (*P* < 0.001).

Dixon et al.,^74^ in an earlier study of the Onduo VDC program, investigated changes in HbA1c among 740 T2D adults treated with basal insulin only, basal insulin plus noninsulin medications, and noninsulin medications.^74^ Participants were stratified by baseline HbA1c, >9.0%, 8.0%–9.0%, and 7.0%–<8.0%. At mean 4.2 months follow-up subsequent to receiving their first rtCGM sensor, participants experienced significant reductions in HbA1c, with the greatest reductions observed in the >9.0%, (−2.3% ± 1.9%), followed by those in the 8.0%–9.0% (−0.7% ± 1.0%) and 7.0%–<8.0% (−0.2% ± 0.8%) groups (all *P* < 0.001).

## Discussion

In 23 (79%) of the 29 studies reviewed here, investigators reported associations between CGM use and improvements in HbA1c^44–47,54,56–60,62,63,65,67,69,71,75^ and/or key CGM metrics.^44,47,71^ These findings, alone, provide strong evidence that supports providing access to this technology to all less-intensively treated T2D patients who would benefit and are able to use it safely and effectively. Monitoring regimens (persistent or intermittent) should be based on each person's individual needs and modified when these needs change.

Although findings from the randomized trials of persistent CGM (rtCGM or isCGM) use demonstrate its superiority over traditional BGM,^44–47^ they do not reflect the actual value and utility of CGM given the high rates of poor adherence to prescribed BGM regimens.^18–22^ Although not well studied, one can reasonably assume that poor BGM adherence is a likely contributor to the high prevalence of poorly controlled diabetes in the United States,^2,6–10^ which continues to worsen.^2^

By design, current CGM systems address all of the most common reasons reported by patients for not performing BGM.^19^ As demonstrated in the Onduo T2D studies, satisfaction was extremely high, users found their experiences with CGM both helpful and enlightening,^69^ and diabetes-related distress was significantly reduced.^72^ Moreover, the rapidly increasing adoption of CGM, worldwide, is a strong indicator of the value patients with diabetes place on this technology.

While some may argue against expanding access to CGM for economic reasons, it is important to consider the costs of uncontrolled diabetes. In 2017, the estimated total cost of diabetes in the United States, included ∼$237 billion attributable to direct medical care and an additional $90 billion in reduced productivity.^9^ A substantial percentage of these costs results from hospitalizations and emergency department utilization subsequent to diabetes-related adverse events.^10^ Most of these events are avoidable. Moreover, as demonstrated in an early study by Fonda et al.^66^ and more recently by Norman et al.,^54^ use of CGM by patients treated with nonintensive therapies is cost effective.

Both public and commercial health insurers are now recognizing the challenges and complexity of effective diabetes management and how current and evolving technologies such as CGM, sensor-augmented insulin pump devices and now, and automated insulin delivery systems can dramatically reduce patient burden and the increasing costs associated with uncontrolled diabetes. However, payers need to have a more comprehensive calculation of the actual cost:benefit ratio, recognizing that the anticipated cost savings of not utilizing these technologies is far outweighed by the unanticipated consequences, such as increased hospitalizations and emergency department utilizations, which are associated with suboptimal glycemic control. This will require greater communications between the various departments within each payer organization to fully assess how the coverage policy will impact the total costs of providing or not providing coverage.

One example of how working with incomplete data can obfuscate the true cost of a policy decision is how the Centers for Medicare & Medicaid Services (CMS) interpreted the “cost savings” attributed to its national launch of the competitive bidding program in 2013. A 2017 study by Puckrein et al., examined changes in accessing BGM supplies test strips between 2010 and 2014, before and after nationwide expansion of the competitive bidding program.^76^ They found that the proportion of beneficiaries filling their test strip prescription partially or not at all grew significantly during that period.

In a 2016 study that looked only at the test period for competitive bidding, a correlation between lower rates of BGM use and increases in mortality, inpatient admissions and costs were observed.^77^ This demonstrates that coverage decisions regarding CGM and other innovative technologies must involve all stakeholders from payer organizations as well as the broader diabetes community.

CMS took an important step on April 16, 2023, expanding coverage of CGM to users of any insulin as well as to individuals who do not use insulin but who experience recurrent Level 2 or a single Level 3 hypoglycemic event.^76^ These changes are consistent with current American Diabetes Association (ADA) clinical guidelines.^37^ In light of the evidence reviewed here, there is clearly a growing evidentiary basis for permitting others to access this important technology as well.

Given the growing prevalence of T2D in the United States, particularly in younger patients, clinicians and health care systems will face multiple challenges to provide quality care to patients who will be living longer with their disease, which will significantly increase their risk for debilitating and costly diabetes complications.^78–80^ Overcoming these challenges can only be achieved when patients and clinicians have unfettered access to the tools and technologies that have been proven effective in improving diabetes management and engaging patients in their self-management regimens. The more individuals understand their diabetes, the more willing and better able they will be to perform the daily tasks required to achieve optimal diabetes self-management.

## Conclusions

CGM should be made readily available to all individuals with diabetes who are able to use this technology safely and effectively. Large randomized controlled trials and prospective real-world studies assessing the benefits of CGM compared with BGM in real-world settings would be helpful. Payers and policy makers need to catch up to the current research, broadening coverage and eliminating preauthorization requirements. Researchers need to catch up to needs of most people with T2D who currently have limited access to CGM and its benefits.
